# P-104. Impact of Sepsis-Induced Cardiomyopathy on Clinical Outcomes in Sepsis Patients

**DOI:** 10.1093/ofid/ofae631.311

**Published:** 2025-01-29

**Authors:** Tae Wan Kim, Ryoung-Eun Ko

**Affiliations:** Chung-Ang University Hospital, Seoul, Seoul-t'ukpyolsi, Republic of Korea; Samsung Medical Center, Seoul, Seoul-t'ukpyolsi, Republic of Korea

## Abstract

**Background:**

Despite the known impact of cardiac function on sepsis outcomes, there is limited understanding of how ejection fraction (EF) influences hospital mortality in patients with sepsis-induced cardiomyopathy. This study assesses the severity of cardiac dysfunction determined by echocardiography within the first 24 hours of sepsis diagnosis on hospital mortality.

**Methods:**

In this retrospective cohort study, 2,504 patients diagnosed with sepsis and who underwent echocardiography within 24 hours were analyzed. Based on EF, patients were classified into normal (EF ≥ 50%), moderate dysfunction (30% ≤ EF < 50%), and severe dysfunction (EF < 30%). The primary outcome was hospital mortality. Multivariable logistic regression was employed to identify factors associated with increased mortality, adjusting for clinically and statistically significant variables.

**Results:**

Among the study patients, 1,803 (72%) had a normal EF, 356 (14.2%) had moderate dysfunction, and 115 (4.6%) had severe dysfunction. The hospital mortality was significantly higher in the severe dysfunction group at 40.0%, compared to 29.5% in moderate and 25.7% in normal EF groups (P=0.023). In multivariable analysis, severe cardiac dysfunction, inappropriate initial antibiotic therapy, and certain sites of infection were significantly associated with increased hospital mortality.

**Conclusion:**

The decreased EF among sepsis patients is correlated with increased organ failure and higher mortality rates compared to patients with normal cardiac function.

**Disclosures:**

**All Authors**: No reported disclosures

